# “That Robot Stared Back at Me!”: Demonstrating Perceptual Ability Is Key to Successful Human–Robot Interactions

**DOI:** 10.3389/frobt.2019.00085

**Published:** 2019-09-06

**Authors:** Masaya Iwasaki, Jian Zhou, Mizuki Ikeda, Yuki Koike, Yuya Onishi, Tatsuyuki Kawamura, Hideyuki Nakanishi

**Affiliations:** ^1^Department of Adaptive Machine Systems, Osaka University, Osaka, Japan; ^2^Kyoto Innovation, Inc., Kyoto, Japan

**Keywords:** robotic salesperson, field trial, multimodal conversation analysis, social presence, situation awareness

## Abstract

Communication robots, such as robotic salespeople and guide robots, are increasingly becoming involved in various aspects of people's everyday lives. However, it is still unclear what types of robot behavior are most effective for such purposes. In this research, we focused on a robotic salesperson. We believe that people often ignore what such robots have to say owing to their weak social presence. Thus, these robots must behave in ways that attract attention encouraging people to nod or reply when the robots speak. In order to identify suitable behaviors, we conducted two experiments. First, we conducted a field experiment in a shop in a traditional Kyoto shopping street to observe customers' real-world interactions with a robotic salesperson. Here, we found that the first impression given by the robot had a crucial influence on its subsequent conversations with most customer groups and that it was important for the robot to indicate it could understand how much attention customers were paying to the robot in the early stages of its interactions if it was to persuade customers to respond to what it said. Although the field experiment enabled us to observe natural interactions, it also included many external factors. In order to validate some of our findings without the involving these factors, we further conducted a laboratory experiment to investigate whether having the robot look back at the participants when they looked at it increased their perception that the robot was aware of their actions. These results supported the findings of the field experiment. Thus, we can conclude that demonstrating that a robot can recognize and respond to human behavior is important if it is to engage with people and persuade them to nod and reply to its comments.

## Introduction

In recent years, several attempts have been made to integrate robots that can communicate with people into different aspects of daily life (Shiomi et al., 2006; Yamazaki et al., 2008; Gehle et al., 2014) because robots are seen as more engaging than animated characters and are perceived as more credible and informative as well as more enjoyable to interact with (Kidd and Breazeal, 2004). Many studies have considered ways to utilize these types of robots. For example, experiments have been conducted on the use of guidance robots in museums (Shiomi et al., 2006; Lee et al., 2010; Tanaka et al., 2015); further, robots have been adopted for educational purposes (Gehle et al., 2014). Additionally, much research has focused on employing robotic salespeople in real-world shops. For example, several studies have shown that specific robot motions have a large influence on people's impressions of the robots (Kanda et al., 2001; Sidner et al., 2004, 2005; Ham et al., 2011), while other researchers have attempted to find particular robot behaviors that attract customers' attention (Yamazaki et al., 2008, 2009). In addition, several researchers have developed robots that can recognize human social behaviors and take advantage of these to attract attention (Gaschler et al., 2012; Das et al., 2015; Fischer et al., 2015). However, these types of behaviors are not always effective in different aspects of daily life. Some researchers developed a robot that can recognize social behavior recognition of human and attract the attention depending on typical social behaviors of human (Gaschler et al., 2012; Das et al., 2015; Fischer et al., 2015). However, these kind of behaviors are not efficient in every aspects of daily life.

In this research, we focus on the behaviors of a robotic salesperson. When there are foreign travelers in a shopping mall, the salespeople in the mall may not be able to speak their language. In such cases, robotic salespeople could help to serve customers, but they are easily ignored by customers due to their lack of social presence, making it difficult for them to work as salespeople. The robots' behavior should draw human attention to them and encourage customers to listen carefully to what they have to say.

In this paper, our goal is to investigate these types of behaviors of robotic salespeople. First, we conducted a field experiment in a shop in a traditional Kyoto shopping street in order to identify behaviors that could draw people's attention to the robot. In this experiment, although we observed natural, real-world interactions between the robotic salesperson and the customers, there were also many external factors. In order to validate some of our experimental findings without involving these extraneous factors, we also conducted a laboratory experiment to examine whether demonstrating the robot's ability to perceive how much attention people were paying the robot could encourage them to respond to its comments.

## Related Work

### Field Trials

Many real-world experiments have already attempted to study natural interactions between robots and humans. For example, experiments have been conducted in museums (Bennewitz et al., 2005; Kuno et al., 2007; Yamazaki et al., 2008; Gehle et al., 2014) and a classroom (Tanaka et al., 2015). In addition, several experiments have employed robots as salespeople for different purposes (Lee et al., 2012; Nakagawa et al., 2013; Niemelä et al., 2017a,b). Two of these experiments were conducted in a shopping mall (Kanda et al., 2010; Shiomi et al., 2013). The first one aimed to use a robot to build customer relationships, while in the second one a robotn offered customers product coupons to improve product sales. However, the robots in these experiments did not introducepresent products to the customers directly. By contrast, in this research we would develop a robotic salesperson that can introduce customers to products in a real shop.

### Attracting Customers to Robots

Many experiments have also been conducted into attracting customers' attention to robots. For example, it was found that tracking customers' faces and head movements could attract their attention in a museum (Yamazaki et al., 2008, 2009). However, that robot was automated and could not communicate naturally with customers. In another study, they placed a robot in an information kiosk to encourage customers to communicate with the robot (Lee et al., 2010), but did not generate a large dataset. In our research, we used remotely controlled robots and conducted two long-term experiments to investigate how to attract and communicate with customers.

## Field Experiment

Robotic salespeople's comments tend to be easily ignored due to their weak social presence, meaning that they may not be effective. The robots' actions must therefore attract human attention and encourage people to listen to what robots have to say. In this section, we conduct a field experiment in order to identify robot behaviors that can draw people's attention to it by observing its natural, real-world interactions with customers. The fact that the robot is not-ignored means that the customer responds continuously to the robot's speech. That is, two-way conversation is established. In this section, we focus on the robot's initial utterances, drawing on previous research suggesting that first impressions are important in human–human interactions (Kelley, 1950), then investigate how to establish two-way conversations.

### Method

#### Experimental Setup

**Figure 1:**
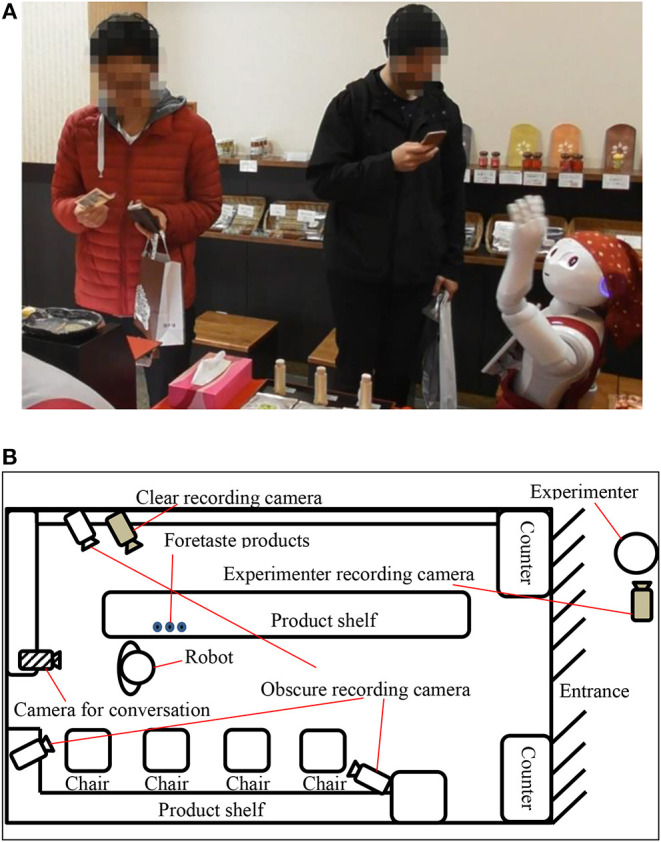
Setup of the field experiment, showing **(A)** a photograph, and **(B)** a top-down view of the shichimi shop.

We conducted the experiment in a shichimi (seven-spice blend) shop located in a traditional Kyoto shopping mall. Figure 1A shows a photograph of the shop, where we installed a Pepper robot as a salesperson. We used Pepper because it has many sensors, enabling us to easily obtain real-time data from the customers, as well as a robot-mounted tablet that we could use to show them pictures of the products. We developed a remote controler and installed a predetermined set of actions in the robot before the experiment began.

Here, we used the Wizard of Oz (WOZ) method (Saerbeck et al., 2010) to control the robot remotely, with an experimenter selecting appropriate reactions for Pepper based on the current situation. With the WOZ method, it takes time for the operator to determine the robot's next behavior and implement it, but this is not a serious problem when the robot is talking with visitors. Pepper's behaviors were divided into two types: ordinary conversation and product introduction. Its ordinary conversation behaviors included greetings (such as “Hello”), handshake requests, and self-introduction, while its product introduction behaviors included offering customers a sample to try, trying to promote sales of shichimi and soft-serve ice cream, and asking a salesperson for help. When offering customers a sample, Pepper would point to the sample's location with its left hand and say, “Would you like to try a sample? You can taste here.”

We placed a camera behind the robot to enable the experimenters to observe the situation in the shop and choose its next action. The robot could also turn its head automatically to focus on people's faces using a camera on its head. To record data, we set up three obscure recording cameras in the shop, as shown in Figure 1B. Here, the customers' faces have been obscured. We also set up one clear recording camera in the shop. When customers approached the robot, they were shown a consent form on the tablet. Only when they had given their consent did we begin recording with the camera. We also placed some handouts on the robot's leg that gave further information about the whole experiment. This experiment was approved by Osaka University's Research Ethics Committee.

#### Analysis Method

In order to observe and analyze the structure and patterns in the robot's interactions with customers, we conducted a multimodal conversation analysis. First, we transcribed the conversations with each group in detail based on the acquired video footage. In addition to the words spoken, the transcripts also described the timing of the customers' remarks, as well as their body movements, gaze direction, and so on. Then, we used these transcripts and videos to analyze the interactions, taking into account both verbal and non-verbal information. Here, we defined a customer group as a group of people who knew each other and entered the shop at the same time, determining this by using the video to confirm that they entered the shop together and talked to each other.

### Results and Discussion

This experiment was carried out over 10 days in 2017. During this time, around 360 customers visited the shop, divided into 164 groups with an average of 2.2 people per group.

In order for a robotic salesperson to offer services to customers and encourage them to make purchases, it needs to attract their attention to what it has to say. Thus, it was vital to investigate which types of action the robot could use to attract the customers' attention. When Pepper received two or more consecutive replies from the same customer, we defined it as a two-way conversation. However, if the customer either did not respond or only replied once, we defined it as a one-way interaction. When we looked for these two types of conversation in our experimental data, we found that 45 groups engaged in two-way conversations, compared with 119 groups whose interactions were one-way. These results suggest that the robot was ignored by most customers.

#### Customers' First Impressions of the Robot Strongly Influenced Their Conversations

In society, robots are generally perceived as mechanical beings that are merely tasked with executing human orders accurately. However, unlike industrial robots, some robots now coexist with people in everyday society. Thus, the relationships between humans and robots should not only involve humans giving commands to robots, but also robots being able to communicate interactively with humans on an equal footing. In this section, we investigate which of the robotic salesperson's behaviors persuaded customers to respond to its comments.

A previous study of human–human interactions found that people's behavior toward others is shaped by their first impressions, with people who have favorable first impressions of someone tending to interact more with them than others who have formed unfavorable impressions (Kelley, 1950). Although that research focused on human–human interactions, this finding may also be applicable to human–robot interactions, so we focused on the robot's initial utterances and examined how best to establish two-way conversations.

First, we compare the group that had a two-way conversation with the group that robot spoke one-way utterances.

Transcript 1 **The group that had a two-way conversation with Pepper (December 5th 16:17:17-16:18:26)**

**Table:** 

1		((C1 looks at Pepper))
2	P	Hello! =
3	C1	Hi!
4	C2	Hello
5	P	My name is Pepper.
6	C1	Hi, Pepper!
7	P	Nice to meet you.
8	C2	Nice to meet you too.
9	C1	Nice to meet you too. (1.0)
10	C1	Hi, Pepper::.
11	P	May I shake hands with you?
12	C1	Sure! Hi! Hello! ((C1 is shaking hands with Pepper))
		(…)
19	P	Would you like to try a sample? You can taste here.
20	C1	OK! ((C1 looks at the tasting sample))
(()) : Supplementary explanation · Speaker's behavior		
! : Lively tone		
= : Speech and utterance are connected without interruption		
(number) : The length of silence		
“:”: Stretched sound		
(…) : Omission		
(P = Pepper, C1 = young woman1, C2 = young woman2)		

**Figure 2:**
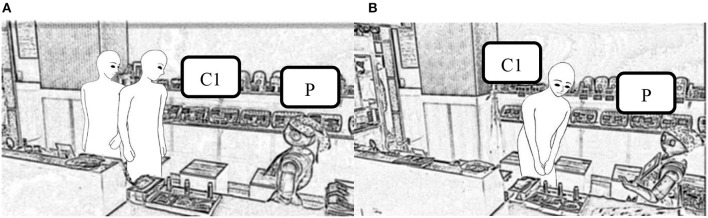
Scenes from Transcript 1, showing **(A)** the robot saying “Hello!” (Line 2), and **(B)** the customers looking at the samples (Line 20).

Transcript 1 shows an example of customers having a two-way conversation with Pepper. At the beginning, when they had just entered the shop (Figure 2A), Pepper said “Hello!” (Line 2), to which the customer replied “Hi!” (Line 3). After that, Pepper made some brief comments, to which the customers replied “Nice to meet you,” (Line 7) and “May I shake hands with you?” (Line 11). We can therefore say that they engaged in a two-way conversation. Once the conversation had begun, even though the robot made slightly longer comments, such as “Would you like to try a sample? You can taste here,”(Line 19) the customer answered “OK!” and looked at the samples (Line 20). Figure 2B shows C1 looking at the samples.

Transcript 2 **The group that robot spoke one-way utterances (August15th 15:59:33-16:00:50)**

**Table:** 

1		((Looking at the products))
2	P	Medium hot shichimi is standard spicy for normal use.
3	P	Very hot shichimi is characterized by a numbing and exciting spicy taste.
4		((C1, C2 and C3 are looking at the products))
5	P	Hello.
6	P	My name is Pepper.
7		((C1, C2 and C3 get away from Pepper))
8	P	Wait, wait. Come on! Let's talk together.
(P = Pepper, C1 = man1, C2 = man2, C3 = man3, S = salesperson)		

**Figure 3:**
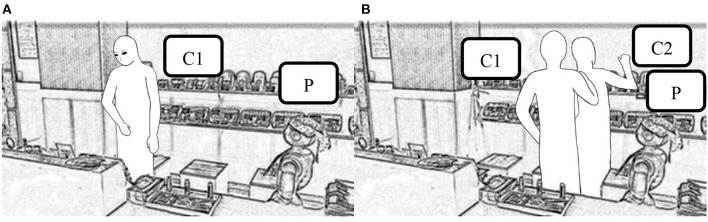
Scenes from Transcript 2, showing **(A)** the robot saying a lengthy explanation (Line 2), and **(B)** the robot being ignored (Line 6).

However, Transcript 2 gives an example of a one-way interaction. When the customers entered the shop, Pepper gave a lengthy description of the shop's products, saying “Medium hot shichimi is standard spicy for normal use.” (Line 2, Figure 3A), but they did not respond. Here, we can see that once the one-way interaction had begun, even when the robot made short and easy-to-answer comments, such as “Hello!” (Line 5) and “My name is Pepper” (Line 6), it was simply ignored (Figure 3B).

Comparing these two examples, we see that once the customers had responded to Pepper's comments, the subsequent conversation became two-way. By contrast, when they did not respond to Pepper's comments, the subsequent interaction was one-way. These differences are particularly noticeable at the beginning of the conversation, so the initial impression the robot gives to customers appears to be extremely important, and possibly determines the customers' subsequent attitude to it.

For all the customers who entered the shop, we looked at the robot's first utterance and the customer's initial response. For 31 out of the 35 customer groups that replied to the robot's first utterance (88.6% of cases), this resulted in a two-way conversation. By contrast, only 14 out of the 129 customer groups who did not respond to the robot's first utterance (10.8% of cases) went on to have a two-way conversation. We also validated these results using chi-squared tests, finding that the difference between the two conditions was significant *(x*^2^ = *83.5, p* = *0.0063* × *10*^*–17*^ < *0.05)*. Consequently, we believe that the initial impression given by the robot had a crucial influence on the subsequent conversation for most customer groups.

Among the 14 groups that did not initially respond to Pepper but then went on to have interactive conversations, this was mostly due to the robot using the wrong language or the customers not paying attention to its first comment. In these cases, when the robot said something later, most of the customers were surprised and responded willingly. In addition, four groups replied to the robot's first utterance but then let the interaction become one-way. However, in these cases, the customers included words that seemed to be spontaneous like “I was surprised.”

**Figure 4:**
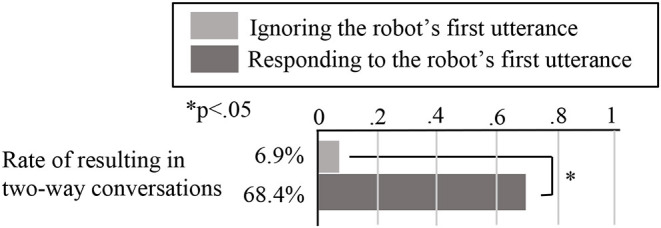
Influence of an initial interaction on the following interaction.

So far, it is unclear whether comment length is all that matters, or whether the content is also important. We therefore compared the interactions in cases where the robot's first utterance was the same, namely “Hello,” which was its most frequent initial comment. The results are shown in Figure 4. For 23 out of the 29 customer groups that replied to the robot's initial greeting, this resulted in a two-way conversation (79.3%). By contrast, only 5 out of the 39 customer groups who did not respond to the robot's greeting went on to have a two-way conversation (12.8%). The results of our chi-squared tests showed that the difference between the two conditions was significant (*x*^2^ = *30.36, p* = *0.03* × *10–6* < *0.05)*.

Given the above, it is reasonable to assume that the impression given by the robot at the beginning of the interaction had a decisive influence on the subsequent conversation for most customer groups. Essentially, the customers' impressions of the robot were determined at the start of the interaction. If they initially perceived the robot as being similar to a voice guidance machine, its subsequent actions tended to be ignored, resulting in a one-way interaction. However, if the customers initially saw the robot as being capable of two-way dialogue, they were much more likely not to ignore its subsequent actions, resulting in a two-way conversation.

#### Establishing the Two-Way Conversation

Having found that it was important for the robot to persuade customers to reply to its first utterance if it was to establish a two-way conversation, we investigated how to encourage customers to reply to the robot. Here, we focused on its initial interactions with customers and compared two customer groups, one where the robot was unable to start a conversation and another where it could.

Transcript 3 **The robot did not start a conversation with the customers(16th August 14:31:07-14:33:54).**

**Table:** 

1		((Entering the shop))
2		((Looking at the products))
3	P	Would you like to try a sample? You can taste here.
4		(3.3)
5	P	Welcome. Please feel free to watch the products.
6	P	Are you troubled to select?
7	P	May I shake hand with you?
(P = robot, C1 = old man, C2 = old woman)

Transcript 4 **The robot started a conversation with the customers(10th April 15:52:45-15:57:30).**

**Table:** 

1		((Entering the shop))
2		((Looking at the products))
3	P	May I help you?
4		((C1 turns his head to look at the robot)) (0.5)
5	P	Hello!
6	C2	Hello↑!
7	C1	Hi::!
8	P	My name is Pepper.
(P = robot, C1 = man, C2 = woman)		

**Figure 5:**
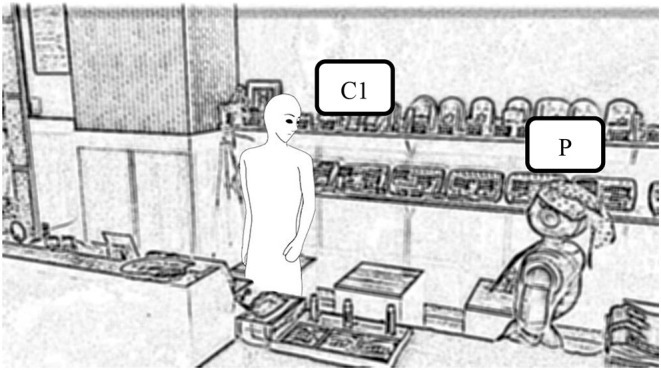
The customer turns his head to look at the robot (Line 4 of Transcript 4).

In Transcript 3, the robot suggested that the customers try a sample (Line 3), but they were looking at the products and did not reply. We believe this was because they did not know whom the robot was speaking to. In Transcript 4, the robot said “Hello!” (Line 5) while the customers were looking at it (Line 4, Figure 5). In that case, the customers replied to the robot (Line 6), and we believe this was because the robot greeted them while they were looking at it. Thus, they realized that the robot was talking to them, establishing a state of mutual perception.

We also wanted to discover whether the robot had to greet customers quickly when they turned their heads to look at it. In Transcript 5, the customer turned her head to look at the robot, but it was slow to greet them: for 3.6 s, she was looking at the robot but it took no action (Lines 3 and 4), and then she turned her head away to look at the products (Line 5). Thus, we believe the robot must greet customers quickly when they turn their heads to look at it, otherwise they will rapidly lose interest.

Transcript 5 **The customer turns his head to look at the robot(16th August 15:47:27-15:50:49).**

**Table:** 

1		((Entering the shop))
2	P	May I help you?
3		((C1 turns her head to look at the robot))
4		(3.6)
5	P	(C1 turns her head to look at the products)
6	P	Nice to meet you!
(P = robot, C1 = old woman)		

From the above, when customers turn to look at the robot, that is a good time for it to greet them. Engaging with them at such moments helps them to believe that the robot is aware they have turned their heads. We therefore investigated all the customer groups to see whether they responded to the robot's utterances. Of the 98 customer groups who were looking at the robot when it greeted them, 78 responded (79.6%). By contrast, only 8 out of the 66 customer groups who were looking elsewhere responded (12.1%). Thus, we can see that most of the customers who responded to the robot were looking at it when it greeted them.

**Figure 6:**
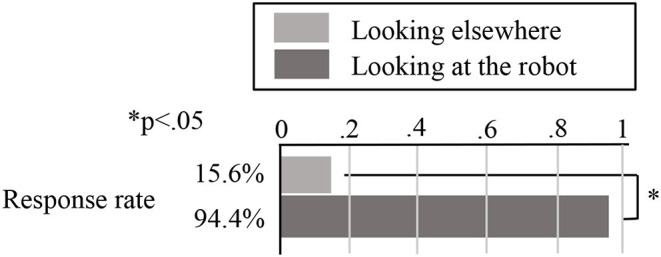
Result of the customers' response.

Our chi-squared test results show that the difference between the two conditions was significant *(x*^2^ = *71.9, p* = *0.021* × *10*^*–15*^ < *0.05)*. However, this does not account for differences in the content of the robot's first utterance, so we conducted another chi-squared test for just the groups where the robot's first utterance was “Hello!” The results, shown in Figure 6, indicate that 34 out of the 36 customer groups who were looking at the robot when it greeted them responded to it (94.4%), compared with only 5 out of the 27 customer groups who were looking elsewhere (15.6%). Again, we can see that most of the customers who responded to the robot were looking at it when it greeted them, and our chi-squared test results show that the difference between the two conditions was significant *(x*^2^ = *43.02, p* = *0.054* × *10*^*–13*^ < *0.05)*.

If the robot responds to customers the moment they see it, this suggests that it is able to perceive the customers' behavior and degree of attention. As a result, customers are more likely to respond to the robot. Essentially, when it shows its perceptual ability to customers, its conversations with them are more likely to be interactive.

## Laboratory Experiment

In the field experiment (Field Experiment), we found that giving the impression that the robot could recognize human behavior encouraged customers to reply. However, since this result was derived from a field experiment, there were many external factors. In order to validate some of our experimental findings without involving these extraneous factors, we also conducted a laboratory experiment to examine whether demonstrating the robot's ability to perceive how much attention people were paying it could encourage them to respond to its comments.

### Hypothesis

In this experiment, we investigated which robot's behaviors persuaded people to respond to its utterances. We believed that it needed to give the participants the impression that it could understand its surroundings, including how much attention they were paying to it, by responding to their non-verbal information. To test this, we adopted a looking-back behavior, where the robot would look back at the participants as soon as they turned their heads to look at it. We then examined whether invoking this looking-back behavior before the conversations began could capture the participants' subsequent attention and encourage them to respond to the robot. Here, we considered the following two hypotheses.

**Hypothesis 1:** The robot's looking-back behavior increases the participants' perception that it is looking at them.

**Hypothesis 2:** The robot's looking-back behavior encourages the participants to respond to it.

### Method

#### Experimental Setting

**Figure 7:**
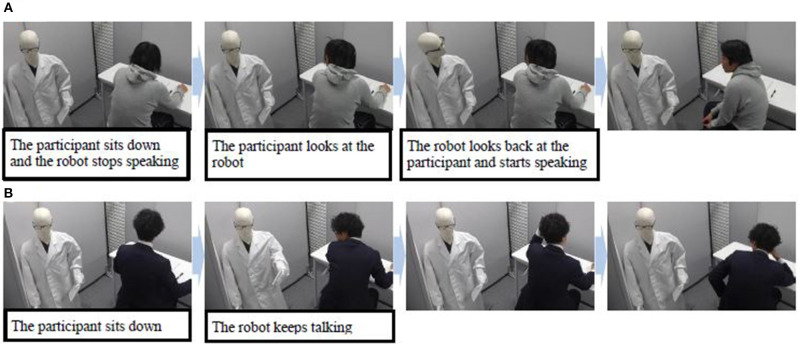
Experimental procedures under both the **(A)** looking-back and **(B)** no looking-back behavior conditions.

**Figure 8:**
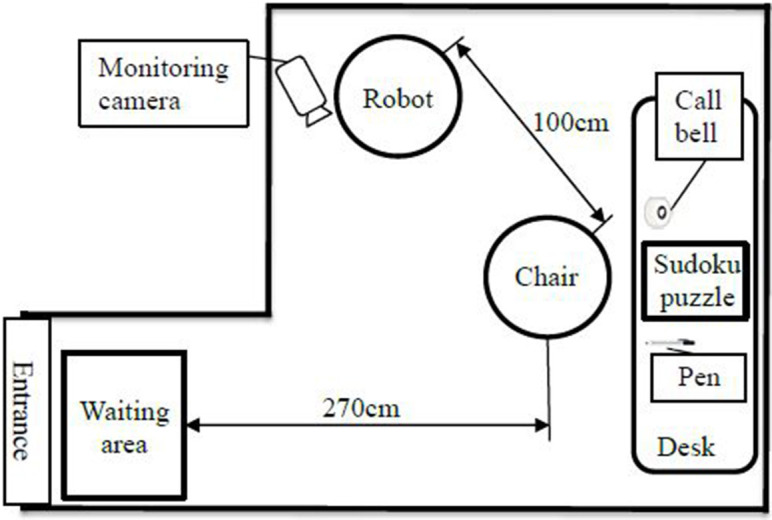
Overview of the laboratory experiment.

For this experiment, we adopted the simply designed robot shown in Figure 7. We did not add features such as eyes, a nose, or a mouth to the robot's face because we suspected that its expression might influence the participants' impressions of it. However, we did make the robot wear glasses to show the direction of its line of sight. Figure 8 shows the experimental setup. We placed the robot behind the participant's chair because we assumed that robots would talk to customers from different directions in real-world shops. This meant that, in order to see the robot, the participants had to turn their heads first.

As a task for the participants to complete, we chose sudoku, an easy logic-based number-placement puzzle, because there was plenty that the robot could say about sudoku puzzles. This experiment was based on an experiment plan that was approved by Osaka University's Research Ethics Committee.

#### Robot Design

**Figure 9:**
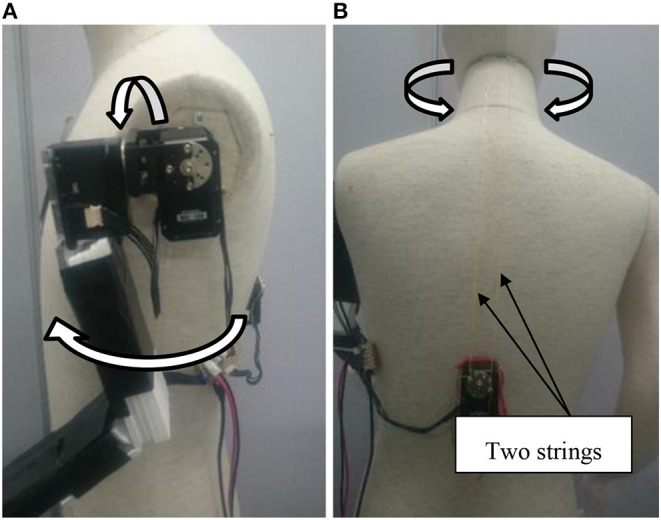
Structure of the robot, showing the **(A)** left arm, and **(B)** neck.

We placed a motor in the robot's shoulder (Figure 9A), enabling it to move its left arm with 2° of freedom, swinging it back and forth and rotating it in and out. In addition, we added a motor to control the neck with two strings (Figure 9B), enabling it to move its head with 1° of freedom, namely left and right. To control the robot remotely, we developed PC-based controler software in advance. During the experiment, we observed the participants and controlled the robot with a camera that we placed beside it. The robot's utterances came from a speaker that we placed behind it, so the participants could locate it based on the direction of its voice, which was synthesized.

#### Procedure

When each participant first entered the experimental room, the robot had already started talking about sudoku. The participant then stood in a waiting area and listened to one of the experimenters explain the following three points about the experiment: the participant was to solve a sudoku puzzle, the robot would signal them when to begin, and the experiment would end when they solved the puzzle. After that, the participant sat down on the chair and waited for the robot to signal them to begin the puzzle. After the robot had talked about sudoku for around 4 min, it gave a signal for the participant to begin. When the participant completed the puzzle, they rang a bell to call the experimenter. After the experiment was over, the participant answered a questionnaire about their impressions of the robot and we discussed their reasons for awarding particular scores and taking the actions they did during the experiment. Finally, the participants were debriefed after the interview.

#### Conditions

To validate the hypotheses (Hypothesis), we focused on one factor and two experimental conditions.

**Factor:** the robot's looking-back behavior

**No looking-back behavior condition:** After the participant entered the laboratory, the robot kept speaking until it signaled them to begin the puzzle.

**Looking-back behavior condition:** After the participant entered the laboratory, the robot kept speaking. However, when they sat down, the robot stopped speaking to show them that it suspected they were not listening to it. After that, when the participant turned their head to look at it, it also turned its head to look at them and resumed speaking until it signaled them to begin the puzzle.

The robot spoke for the same amount of time under both conditions (around 4 min). However, it simply talked about sudoku puzzles in general, and did not include tips or ways to solve the current puzzle, so that the content of its comments did not attract the participants' attention. In addition, the robot's utterances were decided before the experiment. While speaking, its head swung from side to side every few seconds so that it turned toward each participant several times. Its left arm also moved up and down so the participants could see it was a robot when they looked at it. Figure 7 shows the experimental procedures under both conditions. In this example, the participant turned his head to look at the robot even without the looking-back behavior, but not all participants did this. In order to provide a clear understanding of the different conditions in the laboratory experiment, a video is available (Supplementary Material).

#### Participants

Twenty participants (10 females and 10 males) were involved in the experiment. They were all 18–24-year-old university students living in Japan, recruited for the experiment and paid for their contributions. None of them were known to the experimenters. In addition, we used a between-subjects design, because their impressions of the robot under one condition could influence their responses under the other condition.

#### Behavior Evaluation

In this experiment, we first counted the number times each participant responded to the robot's utterances. A participant was seen as responding to the robot if they either made an utterance of their own or nodded without saying anything. Under the looking-back behavior condition, the experimenter observed the participants and made the robot say “Hello” and “Nice to meet you” to them when they turned their heads to look at it. By contrast, under the no looking-back behavior condition, the robot uttered each sentence at predetermined intervals.

We suspected that, if the robot left a wider interval between utterances, it was more likely that the participant would respond, so we analyzed the participants' behavior while keeping the robot's utterances exactly the same under both conditions, during the period when the robot was talking bout sudoku after potentially having looked back. Specifically, we counted the number of responses and measured how long the participants watched the robot when it left equal intervals following each utterance under both conditions. We measured this time based on video recordings taken from the camera shown in Figure 8. In order to examine the changes in the participants' responses over time, we divided the robot's utterances into four parts based on time, splitting its 4 min of speech into four 1-min parts.

Under the looking-back behavior condition, all the participants had to look at the robot so if, during the experiment, the participants did not turn to look at it, we would make the robot say “Please look at me.” However, we felt that this utterance (“Please look at me”) led the participants to look at the robot on purpose so, when we analyzed the experimental results, we also analyzed the data with these cases excluded. In this study, two coders collected the behavioral data and we adopted Cohen's kappa statistic to validate its inter-rater reliability. The results showed that κ values for the participants' responses (κ = 0.79), time spent looking at the robot (κ = 0.80), and number of spoken replies (κ = 1.0) were all above 0.75.

#### Questionnaire Evaluation

After the experiment, the participants filled out questionnaires regarding their impressions of the robot in order to evaluate Hypothesis 1. They responded using a 7-point Likert scale going from 1 (strongly disagree) through 4 (neutral) to 7 (strongly agree), and we also included a free description section. Afterwards, we interviewed the participants about their reasons for awarding particular scores and acting as they did during the experiment. The questionnaire included the following seven questions. Here, Q1 assessed the quality of the robot's speech, Q2 checked for manipulation, and the remaining questions were related to the participants' impressions of the robot.

Q1. The robot's voice was sufficiently clear.
Q2. I felt I was being observed by the robot.
Q3. I felt the robot was waiting for my reply.
Q4. I felt the robot's behaviors were similar to human ones.
Q5. I felt I was being forced to listen to the robot.
Q6. I felt I was being forced to respond to the robot.
Q7. I felt the robot was reacting to my behavior.

### Results and Discussion

#### Results

**Figure 10:**
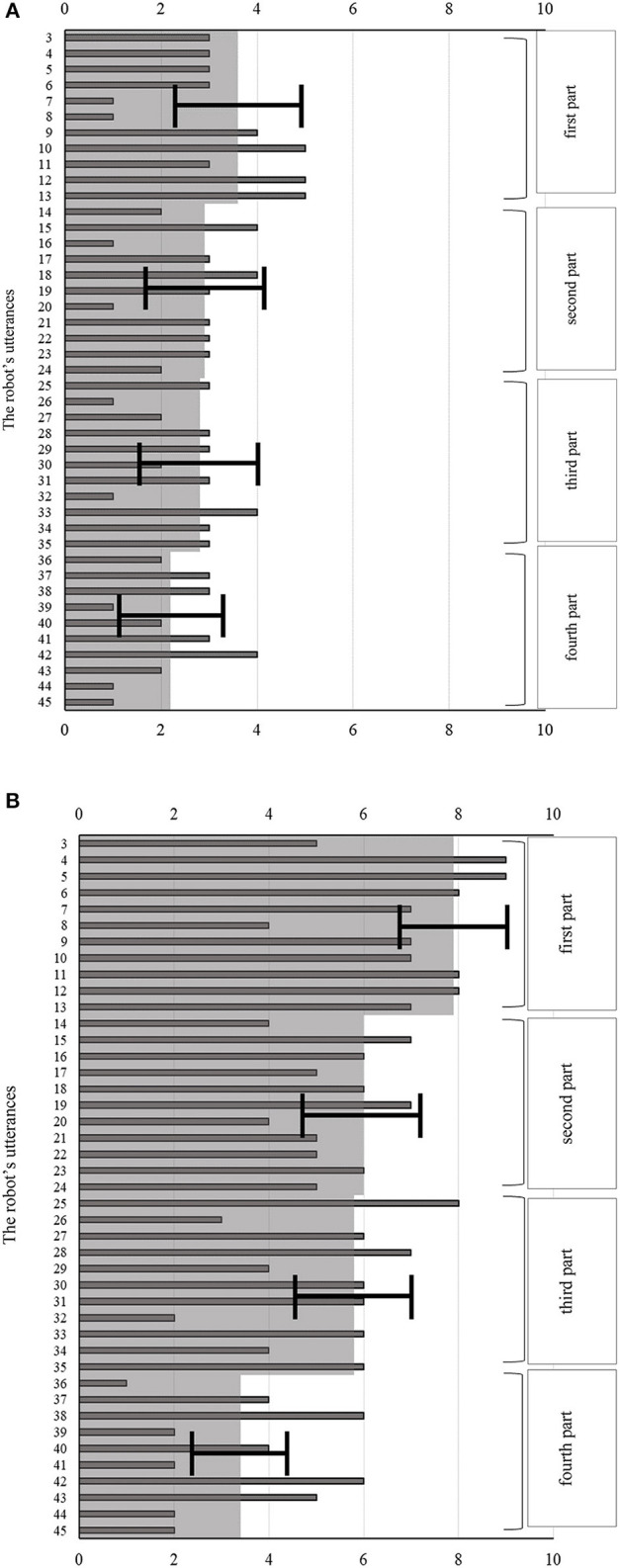
Histograms representing the number of responses to each of the robot's utterances, under the **(A)** no looking-back, and **(B)** looking-back behavior conditions. Here, the bars represent the average numbers of responses, while the light gray shaded areas represent the overall average numbers of responses for each part and the error bars represent the standard errors.

**Figure 11:**
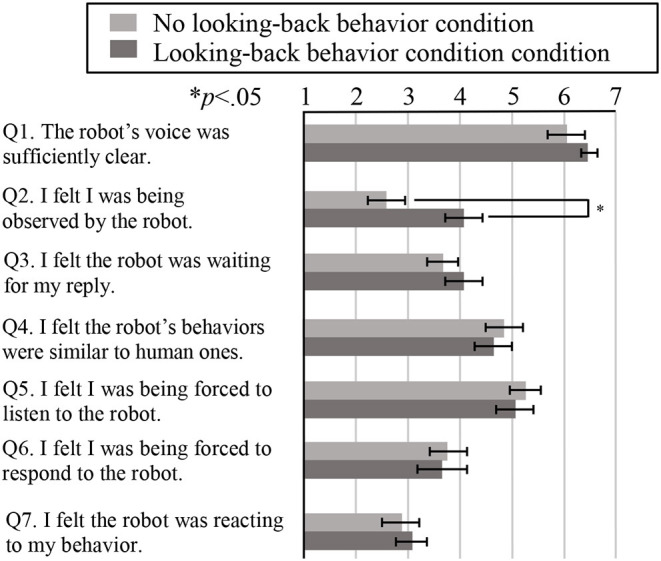
Questionnaire evaluation results. Here, the bars represent the average scores for each question, while the error bars represent the standard errors.

Figure 10 shows the behavior evaluation results, and Figure 11 shows the questionnaire results. For these analyses, we carried out two-tailed independent *t*-tests. For the speech quality question (Q1 in Figure 11), we found no significant difference between the two conditions, so we believe that the robot's speech quality did not influence the participants' behavior or their impressions of the robot. In addition, the results for Q2 (whether the participants felt the robot was observing them) showed a significantly higher score under the looking-back behavior condition than under the no looking-back behavior condition (*t*(18) = 2.11, *p* = 0.049 <0.05, Cohen's *d* = 0.95), supporting Hypothesis 1. There were no significant differences between the two conditions for any of the other questions.

In the behavior evaluation results shown in Figure 10, the difference in the total number of responses across all four parts of the experiment between the two conditions was not significant (*t*_(18)_ = 1.86, *p* = 0.079 <0.1, Cohen's *d* = 0.83). From Figure 10, the more the robot talked, the fewer responses the participants made, under both conditions. We therefore analyzed whether the number of responses during each part differed between the two conditions.

**Figure 12:**
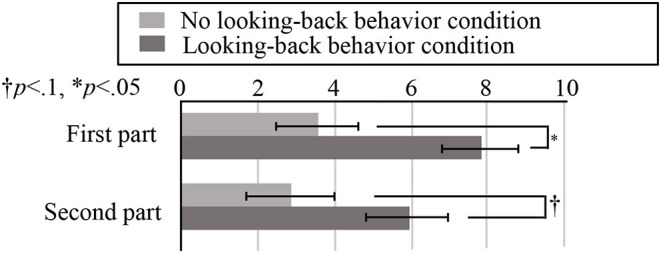
Average numbers of responses during the first and second parts, under both conditions. Here, the bars represent the averages, while the error bars represent the standard errors.

Figure 12 shows the average numbers of responses during the first and second parts. For the first part, we found that the difference was significant (*t*_(18)_ = 2.47, *p* = 0.024 <0.05, Cohen's *d* = 1.10). By contrast, the difference in the numbers of responses during the second part was a non-significant tendency (*t*(18) = 1.77, *p* = 0.093 <0.1, Cohen's *d* = 0.79). Finally, we found no significant differences in the numbers of responses during the third and fourth parts. Thus, Hypothesis 2 was only supported during the early stages of the robot's comments.

#### Discussion

Regarding the questionnaire, the results for Q2 showed that the feeling of being observed by the robot was significantly stronger under the looking-back behavior condition. During the interviews, the participants made comments such as, “When I turned around, I made eye contact with the robot,” and “When I turned my head to the robot, it also looked at me.” This confirms that the participants had the impression that the robot was looking back in response to them turning their heads and looking at it.

From the behavior evaluation results, we see that although there was a difference in the total number of responses to the robot's utterances, it was not significant. We believe this was because the robot's looking-back behavior only occurred at the start of each experiment, so the participants' impression of the robot faded away over time and they stopped feeling that it could understand their behavior. In addition, there was no significant difference between the two conditions in the responses to questions Q5–Q7 on the questionnaire. We believe this was because the participants had much stronger impressions of the latter half of the experiment because they answered the questionnaire afterward. Moreover, when interviewed, one of the participants said that “I felt that my actions were being observed by the robot when it looked back at the beginning, but as time went on, this faded away.”

Given these results, we divided the robot's utterances into four 1-min parts to investigate how the number of responses changed over time (Figure 10). This showed that the more the robot talked, the fewer responses the participants made, under both conditions. When we focused only on the first part of the robot's utterances, there was a significant difference in the number of responses between the two conditions, which we believe is because the robot's looking-back behavior made a strong impression on the participants during this time.

It is also possible that another reason for this was that we did not consider the concept of turn-taking. In a previous study, a robot only looked at people when it was asking them to respond (Chao and Thomaz, 2010). Thus, we might have been able to maintain the number of responses by repeating the robot's looking-back behavior while it was talking. During their post-experiment interviews, most of the participants said that “I wanted to show that I was listening to the robot.” Under the looking-back behavior condition, not only their faces but also often their bodies were turned toward the robot when they were listening to it. Under the looking-back behavior condition, 9 out of the 10 participants turned their bodies toward the robot. By contrast, only 4 out of the 10 participants did the same under the no looking-back behavior condition.

From the above, we concluded that indicating the robot can understand the participants' behavior and mental state is important for increasing its social presence. The robot's initial behavior enhanced their perception of being looked at by it. After that, they would have felt guilty if they had ignored the robot, so they tried to suggest that they were listening to it and, consequently, were more willing to respond to it.

In this experiment, although we investigated the effect of the robot's looking back, we did not study which behaviors would enable it to show that it was aware of how much attention the participants were paying to it. Moreover, the looking-back behavior was performed before the conversations began, so it is possible that the participants simply become bored when the impression created by this behavior faded away. It is probable that the robot could maintain a strong social presence by performing such behaviors several times during the conversation or adopting the previously mentioned approaches considered in related studies (Shiomi et al., 2006; Yamazaki et al., 2008, 2009). We plan to investigate these points in future work.

## General Discussion

From the field experiment, we found that most of the customer groups fell into one of two categories: either the group replied to the robot's first utterance, resulting in a two-way conversation, or it did not, resulting in a one-way interaction. Therefore, we believe that the impression made by the robot at the beginning of the interaction had a crucial influence on the subsequent conversation for most customer groups. The key was to persuade the customers to reply to the robot's first utterance.

We also clarified that the best time for the robot to first talk to a customer is the moment when they turn their head to look at it. Greeting them at this time potentially makes them believe that the robot is aware they have turned their head. Thus, we believe that giving the impression of recognizing human behavior encourages customers to reply. A previous study found that gaze-based feedback can be used to signal the robot's perception, understanding, and attitude toward the communicated content (Allwood et al., 1992), which also supports our conclusion. The laboratory experiment also supported this conclusion. In addition, the robot established eye contact with the customers when they looked at it, and some studies have shown that eye contact has an impact on various cognitive processes (Senju and Hasegawa, 2005; Dalmaso et al., 2017; Xu et al., 2018). We therefore believe that establishing eye contact was also a factor in our results.

Taken together, our qualitative and statistical results lead us to conclude that indicating the robot can understand people's behavior and mental state is important for attracting their attention and makes it easier to persuade people to listen to it.

In this paper, we conducted both a field experiment and a laboratory experiment. The field experiment did not consider customer differences, such as the number of people in the group or their age, gender, or nationality, even though these could have affected their interactions with the robot. We also did not consider the effect of different utterances. We plan to investigate these issues in future work.

## Conclusion

This paper has focused on encouraging customers to respond to a robotic salesperson's initial utterances. With this in mind, we conducted two experiments to investigate the initial stages of human–robot interactions, namely a field experiment and a laboratory experiment, in order to investigate what types of behaviors the robot should adopt and when it should perform them. First, we conducted a field trial to observe natural interactions between a robot and customers in a real shop. Then, we conducted a laboratory experiment to investigate whether having a robot look back at the participant when they looked at it increased their perception that the robot was aware of their actions.

Based on the results, we found that suggesting the robot could recognize human behavior in the initial stages of its interactions with customers made them feel as if it was looking at them and encouraged them to respond to its utterances. Our most important finding is that, in conversations between people and robots, it is important to suggest that the robots are aware of their behavior and state of mind. Such behavior makes people feel that the robot can understand their behavior and respond accordingly, so they are more likely to show they are listening to it. We hope that this research will promote human–robot conversation and enable us to use robots more effectively.

## Ethics Statement

Our experiments have approved from Research Ethics Committee of Osaka University.

## Author Contributions

MIw contributed to design, conduction, data collection, analysis of the both experiments, and writing the paper. JZ contributed to design, conduction, data collection, analysis of the field experiment and writing the paper. MIk contributed to design, conduction, and data collection of the field experiment. YK contributed to design, conduction, data collection, and analysis of the laboratory experiment. YO contributed to design and analysis of the laboratory experiment. TK contributed to design and conduction of the field experiment. HN critically reviewed the paper and gave the final approval of the version submitted.

### Conflict of Interest Statement

TK is the CEO of Kyoto Innovation, Inc. The remaining authors declare that the research was conducted in the absence of any commercial or financial relationships that could be construed as a potential conflict of interest.
